# Monophasic synovial sarcoma of the pharynx: a case report

**DOI:** 10.1186/1477-7800-6-9

**Published:** 2009-03-31

**Authors:** Dibendu Betal, Ramesh Babu, Veysi Mehmet

**Affiliations:** 1 Department of Surgery, St Mary's Hospital, Isle of Wight NHS Trust, Parkhurst Road, Newport, Isle of Wight, PO30 5TG, UK

## Abstract

Synovial sarcomas are a rare form of soft tissue sarcomas. We present a case of a 62 year-old male presenting with a left thyroid lump initially though to be a thyroid adenoma but subsequently diagnosed as a monophasic synovial sarcoma of the pharynx. We discuss the diagnosis and treatment of this case.

## Background

Synovial sarcomas are named as they were believed to originate from the synovium but less than 10% are intra-articular. They are a rare form of soft tissue sarcoma after liposarcomas, malignant fibrous histiocytomas and fibrosarcomas and make up 10% of all sarcomas. They normally involve large joints and lower extremities. They are often small in size with a slow growth and well-circumscribed resembling benign lesions. This case demonstrates a rare occurrence of a synovial sarcoma in the pharynx.

## Case presentation

A 62 year-old gentleman presented to thyroid clinic with a neck lump. It had been growing slowly over a six-month period and became more noticeable. He did not report any hoarse voice, dyspnoea or dysphagia. There was no loss of appetite or weight. On examination there was a palpable well circumscribed lump arising from the left thyroid lobe measuring 5 × 3 cm. There was no tracheal deviation and the lump moved on swallowing. There were no other palpable lymph nodes and the patient was clinically euthyroid.

His previous medical history of note includes a sub-arachnoid haemorrhage ten years previously leaving the patient with residual mild left sided weakness. He was a smoker of ten cigarettes a day and was previously a heavy drinker.

Thyroid function tests were within normal limits and thyroid ultrasound demonstrated a lump that appeared benign arising from the superior pole of the left lobe of thyroid gland so the initial diagnosis was an adenoma in the left lobe of thyroid.

During the operation it was noted that there was a cystic lump that did not originate from the thyroid gland but from the lower pharynx extending to the upper oesophagus. The lump was found to be full of necrotic tissue. The lump and left lobe of the thyroid were excised.

**Figure 1:**
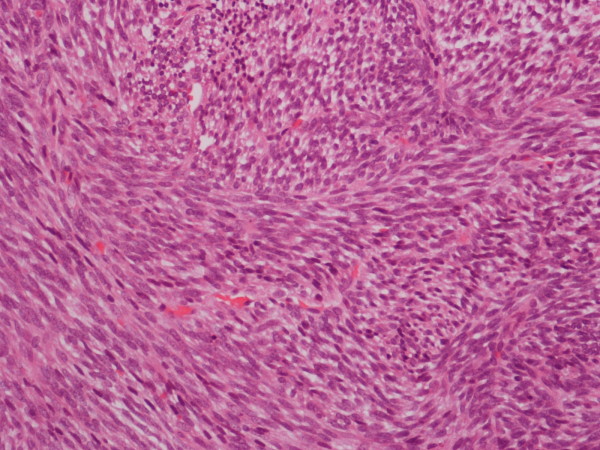
**Monophasic spindle cells**.

The histopathology demonstrated a normal left thyroid gland. The 5 cm tumour was well circumscribed with focally invasive spindle cells (Figure 1) with some areas that were highly cellular with a fascicular fibrosarcomatous like growth pattern. Elsewhere there was rather loose fibrous and partly myxoid stroma with abundant mast cells. In addition immunochemistry including EMA, CD99 and BCL2 were all positive supporting the diagnosis of monophasic synovial sarcoma originating from the lower pharynx.

Staging CT-scan showed local disease recurrence with infiltration of the strap muscles and trachea. He was started on a course of radical radiotherapy. Follow-up CT-scan revealed multiple pulmonary metastases although the patient remains clinically stable.

## Discussion

Synovial sarcomas [1] are named as it was believed that they originate from synovial lining cells although there is little evidence that the anatomical origin is within a joint. They often present in adolescents or young adults with a slight predominance of males. Clinically the features may be non-specific and does not distinguish it from other sarcomas. They often present as a slow growing well-circumscribed mass that mimics benign pathology. In rare circumstances, patients present with symptoms secondary to pulmonary metastases.

With regards to histopathology [2], up to two-thirds of synovial sarcomas often have a biphasic appearance that consists of a dual line of differentiation of tumour cells. They are composed of a mixture of elongated basophilic spindle cells and glandular structures made of columnar epithelial cells. The spindle cell areas resemble fibrosarcoma cells with haemagiopericytoma type vascular pattern that often show small areas of calcification or metaplastic bone. Up to one-third of cases are monophasic in nature that are composed of only spindled cells or very rarely, epithelial cells. However, lesions composed solely of spindle cells may be misdiagnosed as fibrosarcoma or malignant peripheral nerve sheath tumours. Immunochemistry helps identify these tumours as they show positive reactions for keratin and epithelial antigen that differentiate them from other sarcomas. Specific translocation has helped to classify synovial sarcomas as they typically show a t(X;18) translocation with fusion between SSX1 and SYT genes with a biphasic appearance in two-thirds of cases whilst the remainder show a fusion between SSX2 and SYT genes.

The first case reports of synovial sarcoma were reported in knee joints [3-5] and the first literature review of cases was published at the beginning of the 1900s [6]. There have been a few publications relating solely to monophasic synovial sarcoma and these have mainly been in the lung [7-11], and fewer reports in the nerves [12,13], gastrointestinal tract [14], liver [15], vulva [16,17] and conjunctiva [18,19]. Published cases of synovial sarcoma of the pharynx are rare [20-22] and there are only two reports of monophasic synovial sarcoma of the neck [23,24].

The treatment of choice is surgery with or without radiotherapy and chemotherapy. The survival rate at five years varies from 25% to 62% and at ten years range from 10% to 30%. The common areas synovial sarcomas metastasise to include the lung, skeleton and occasionally regional lymph nodes.

Studies suggest that the use of adjuvant radiotherapy following surgery has a beneficial effect [25] however neoadjuvant radiotherapy has yet to show any benefit [26]. The role of neoadjuvant or adjuvant chemotherapy in primary synovial sarcoma survival is inconclusive. Early meta-analysis of 14 randomised controlled trials of doxorubicin-based chemotherapy showed no benefit for overall survival [27] and the French Sarcoma group showed no survival benefit with ifosfamide-based neo-adjuvant or adjuvant chemotherapy, however, radiotherapy significantly improved local recurrence-free survival but not distant recurrence-free survival or overall survival [28]. The Italian randomized cooperative trial showed that a combination of anthracycline and ifosfamide showed a statistical benefit for extremity and girdle sarcomas at median follow-up of 59 months [29] however at the conclusion of the trial after a median follow-up of 89.6 months there was no statistical benefit [30]. A more recent study of ifosfamide-based chemotherapy in primary extremity synovial sarcoma showed benefit for disease-specific survival, distance recurrence-free survival but not for local recurrence-free survival [31]. One study specific to head and neck synovial sarcomas showed higher survival and lower recurrence rates with patients treated with surgery and adjuvant radiotherapy than with surgery alone or a combination of surgery, radiotherapy and chemotherapy [32].

## Conclusion

Synovial sarcomas are a rare form of soft tissue sarcomas. This case represents a rare form of monophasic synovial sarcoma of the pharynx. The gold standard of treatment is complete surgical excision with adjuvant radiotherapy. Controversy still remains regarding the use of neoadjuvant and adjuvant chemotherapy however studies have shown benefits with ifosfamide-based chemotherapy in a select group of patients.

## Consent

Written informed consent was obtained from the patient for publication of this case report and any accompanying images. A copy of the written consent is available for review by the Editor-in-Chief of this journal.

## Competing interests

The authors declare that they have no competing interests.

## Authors' contributions

DB Literature searches, writing the paper. RB Performed surgical procedure. VM Performed surgical procedure and supervised final revision of the article
